# Pain Interference, Resilience, and Perceived Well-Being During COVID-19: Differences Between Women With and Without Trauma Exposure Prior to the Pandemic

**DOI:** 10.3389/ijph.2022.1604443

**Published:** 2022-07-19

**Authors:** Elena R. Serrano-Ibáñez, Carmen Ramírez-Maestre, Gema T. Ruiz-Párraga, Rosa Esteve, Alicia E. López-Martínez

**Affiliations:** ^1^ Faculty of Psychology and Speech Therapy, University of Málaga, Málaga, Spain; ^2^ Instituto de Investigaciones Biomédicas de Málaga (IBIMA), Málaga, Spain

**Keywords:** COVID-19, women health, trauma exposure, pain interference, resilience, well-being

## Abstract

**Objectives:** The aim of this study was to investigate the consequences of the COVID-19 pandemic in women with non-malignant chronic pain, and to determine whether women exposed to traumatic situations prior to the outbreak would be at a higher risk of negative health impacts.

**Methods:** A total of 365 women were divided into three subgroups according to whether or not they had experienced a traumatic event prior to COVID-19. They completed an online survey.

**Results:** Significant differences were found between groups during lockdown: 1) more psychological abuse was experienced by the group of women who had experienced an interpersonal traumatic event prior to the pandemic than in the other subgroups; 2) physical activity levels were higher and scores on pain interference were lower in women in the non-traumatized subgroup than in the other subgroups; 3) pain interference was predicted by pain intensity, decreased social support, and resilience, whereas perceived well-being was predicted by pain interference.

**Conclusion:** Women who had experienced a traumatic event prior to the pandemic suffered worse consequences of the COVID-19 lockdown, particularly greater pain interference, although resilience was shown to both mitigate pain interference and enhance perceived well-being.

## Introduction

On March 14 2020, the Spanish government declared a state of emergency to deal with the spread of COVID-19 pandemic and free movement was limited to essential activities for nearly 2 months. In this setting of the outbreak, recent research has shown an association between the COVID-19 pandemic and significant psychological impacts and interference in patients with CP [1]. In addition, greater self-perceived increases in anxiety and depressed mood due to the lockdown conditions were reported by people with CP than by pain-free individuals [2]. Furthermore, pain treatment during the COVID-19 lockdown has changed because of lack to access to health care [3], and difficulty in receiving medical care has been shown to be a significant predictor of emotional distress [4].

Staying at home during the lockdown could increase exposure to stressful situations, such as psychological or physical abuse, as was recently reported in a study that investigated patterns of abuse in the UK during the COVID-19 pandemic [5]. Moreover, it has been suggested that there is a need to study the specific peri- and posttraumatic implications of this crisis [6]. In addition, previous research has found that physical health is worse in individuals who have experienced interpersonal trauma than in individuals who have experienced non-interpersonal trauma [7]. This finding has also been supported in populations with CP [8]. Patients with comorbid posttraumatic stress symptoms and CP make higher demands on health care, and report worse quality of life than patients with CP alone [9].

Even if the coronavirus pandemic is considered to be a stressful event, personal resilience may help people to effectively manage COVID-19-related stress. Resilience is the capacity to successfully adapt to challenges that threaten the functioning of an individual that affects how people manage traumatic events by reducing their psychological cost [10]. Research on resilience and adjustment to CP has shown that resilience is a protective variable in pain adjustment that increases quality of life in patients with CP [11, 12], even in populations that have experienced trauma events prior to pain onset [12, 13]. Moreover, a novel study showed that psychological flexibility appeared to buffer the impact of the COVID-19 pandemic on daily functioning [1]. However, previous research has also demonstrated that, following a disaster, chronic psychological dysfunction tended to be higher in individuals exposed to more severe pre-stressors than in those who had never experienced one [10], and that posttraumatic stress symptoms due to past traumatic events were associate to pandemic-related peritraumatic stress symptoms, particularly in women [14]. Further, levels of COVID-related anxiety, depression, and stress symptoms were higher in people who had experienced traumatic events before the pandemic than in people who had not experienced such events [15].

Notwithstanding this background, no study could be found that investigated the relationship between past traumatic events and the psychological effects of the pandemic on people with chronic pain. Therefore, the aims of this study were:

(1) To investigate the consequences of the COVID-19 pandemic in women with non-malignant CP. Specifically, we investigated whether women exposed to traumatic situations prior to the outbreak would be at particular risk of negative impacts due to COVID-19 outbreak.
(2) To examine the differences between women who had experienced a traumatic event and women who had never experienced a traumatic event prior to the pandemic in pain impairment, resilience, and perceived well-being. Based on prior research, we expected to found significantly higher levels of pain interference and lower levels of resilience and perceived well-being in women who had experienced traumatic events—in particular, interpersonal traumatic events—prior to the COVID-19 outbreak.
(3) To determine which variables (i.e., the consequences of COVID-19, pain-related variables, and resilience) predicted both scores pain interference and perceived well-being in each group.

## Methods

### Participants and Procedure

Data collection was conducted during the Spanish lockdown between 10 April 2020 and 30 April 2020. The research protocol was developed using LimeSurvey 2.0. A random sample of 198 associations were invited to share the survey link from a total of 250 Spanish CP associations recorded on the website of the Spanish Pain Society and the Spanish Ministry for Home Affairs. People interested in participating voluntarily accessed the link. The first screen of the survey site showed the consent form. Participants could only access the research protocol if they gave their informed consent to participate. The study procedures complied with the Declaration of Helsinki and received institutional review board approval from the University Ethics Committee.

A total of 423 women and 54 men returned the survey. In total, 58 women were excluded from the initial sample: 12 participants did not indicate whether they had experienced a traumatic event or not, and 46 had a diagnosis of oncological pain or did not have CP. Due to the low number of men, we decided to conduct the study by only including the data on women. The inclusion criteria were as follows: 1) being from 18 to 65 years old; and 2) having a non-oncologic CP condition for at least 3 months.

The final sample comprised 365 women divided into three subgroups according to whether or not they had experienced a traumatic event prior to the COVID-19 outbreak: 1) 100 women who had never experienced a trauma (NT group); 2) 130 women who had experienced at least one non-interpersonal trauma (NIT group); and 3) 135 women who had experienced at least one interpersonal trauma (physical and/or sexual abuse) and any other traumatic event (IT group).

### Measures

#### Demographic and Clinical History

All participants were asked to provide information on age, marital status, the highest educational level completed, primary CP diagnoses, and pain duration.

Exposure to past traumatic events. Participants were asked to indicate if they had personally experienced traumas prior to the pandemic. These events were selected from the Life Events Checklist for DSM-5 Standard Version [16].

#### Exposure to Past Traumatic Events

Participants were asked to indicate if they had personally experienced non-interpersonal traumas (i.e., a life-threatening transportation accident and/or an accident at work, home, or during recreational activity; a life-threatening illness or injury; and an unexpected death and/or sudden violent death of a close person) or interpersonal traumas (i.e., physical abuse, sexual abuse, or sexual assault). These events were selected from the Life Events Checklist for DSM-5 (LEC-5) Standard Version [16] according to the most frequent ones among the general population worldwide [17]. A forward-backward translation method was used to adapt this scale into Spanish [18].

#### Variables Related to the COVID-19 Outbreak

Participants provided information on whether they were experiencing any of the following situations during lockdown: 1) having contracted COVID-19, having lived with someone with coronavirus, death of a close person due to COVID-19, changes in access to medical treatment for pain, and loss of employment (response format: Yes/No); and 2) changes in daily life functioning due to the pandemic lockdown: maintaining daily routines (response format: never (score 0) to always (score 5); decreased physical activity and decreased social support (response format: not at all (score 0) to very much (score 5); and 3) being psychologically, sexually, and/or physically abused (response format: Yes/No).

#### Pain Intensity

A rating scale ranging from 0 (no pain) to 10 (worst pain) was used to assess the current, highest, lowest, and average pain intensity over the previous 2 weeks. The mean of these four ratings provided a single composite score of characteristic pain intensity [19]. Cronbach’s alpha coefficient was *α* = 0.88.

#### Pain Interference

Pain interference was assessed using the Spanish version of the Short Form of the Brief Pain Inventory [20]. This instrument assesses pain severity and its impact on daily functioning. It comprises seven items which are scored on an 11-point Likert-type scale ranging from 0 (does not interfere) to 10 (interferes completely). The participants were asked about their pain experience over the previous 2 weeks. Cronbach’s alpha coefficient was *α* = 0.93.

#### Resilience

Resilience was assessed using the Spanish version of the Brief Resilience Scale [21]. Participants were asked to report their ability to recover from stressful circumstances over the previous 2 weeks on a 5-item Likert-type scale ranging from 1 (strongly disagree) to 5 (strongly agree). A higher score indicates a higher degree of resilience. Cronbach’s alpha coefficient was *α* = 0.70.

#### Perceived Well-Being

Perceived well-being was assessed using the Spanish version of the 5-item World Health Organization Well-Being Index [22]. This self-report assesses how respondents have been feeling in the previous 2 weeks on a 6-point Likert-type scale ranging from 0 (never) to 5 (all the time). Cronbach’s alpha coefficient was *α* = 0.84.

### Statistical Analyses

Analyses were performed using SPSS version 25.0. Homogeneity was analysed by calculating differences in demographic and clinical variables between the NT, NIT, and IT groups, using chi-squared tests for categorical outcomes and ANOVA for continuous variables. Chi-squared tests were also calculated to compare groups by categorical variables related to COVID-19. ANOVA was used to compare groups in maintaining daily routines, decreased physical activity, and decreased social support. It was also used to determine differences between the three groups in pain intensity, pain interference, perceived well-being, and resilience. We conducted Bonferroni correction for multiple comparisons.

A total of six univariate GLM were calculated for the three groups of women to determine which variables predicted pain interference and perceived well-being scores in each group. For each group, pain interference and perceived well-being were considered as the outcome variable, and the following variables were considered as predictors in each model: having contracted COVID-19, having lived with someone with COVID-19, death of a close person due to COVID-19, loss of employment due to COVID-19, access to medical treatment, being psychologically abused, maintenance of daily routines, decreased physical activity, decreased social support, pain intensity, and resilience. We also analysed the pairwise interaction effect of the variables that were found to be significant as well as the total interaction effect when appropriate. Pain interference was also introduced in the model to predict scores on perceived well-being.

A *p*-value of less than 0.05 was used as a cut-off for statistical significance. Effect sizes were represented by Cohen’s *d* (reference values of 0.2, 0.5, and 0.8 for small, medium, and large effects, respectively) and by partial eta-square (ŋ^2^_p_) (reference values were 0.01, 0.06, and >0.14 for small, medium, and large sizes, respectively) [23].

## Results

### Sociodemographic Characteristics and Pre-COVID-19 Traumatic Events

Table 1 shows the main clinical characteristics of the three groups of participants, as well as the number of traumatic events experienced by the NIT and IT groups.

**TABLE 1:** Characteristics of the group of women who had never experienced a trauma (*n* = 100), the group of women who had experienced at least one non-interpersonal trauma (*n* = 130), and the group of women who had experienced at least one interpersonal trauma (*n* = 135) (Spain, 2021).

	NT		NIT		IT	
	M (SD)	N (%)	M (SD)	N (%)	M (SD)	N (%)
Pain diagnosis						
Arthrosis		3 (3%)		3 (2%)		0 (0%)
Chronic migraine/headache		4 (4%)		0 (0%)		2 (2%)
Chronic widespread pain		8 (8%)		15 (12%)		17 (13%)
Fibromyalgia		76 (76%)		95 (73%)		102 (76%)
Irritable bowel syndrome		1 (1%)		1 (1%)		4 (3%)
Low back pain		5 (5%)		10 (7%)		12 (8%)
Rheumatoid arthritis		2 (2%)		6 (5%)		1 (0.7%)
Others		1 (1%)		6 (2%)		1 (0.7%)
Length of pain						
Between 3 and 11 months		3 (3%)		3 (2%)		4 (3%)
Between 1 and 5 years		29 (29%)		34 (29%)		56 (29%)
Between 6 and 9 years		17 (15%)		17 (15%)		19 (14%)
10 years or more		51 (51%)		72 (47%)		55 (41%)
Consequences of COVID-19						
Having experienced COVID-19		5 (5%)		3 (2%)		12 (8%)
Having lived with someone with COVID-19		4 (4%)		8 (6%)		9 (7%)
Death of a close person due to COVID-19		8 (8%)		30 (23%)		25 (19%)
Changes in the access to medical care for pain		23 (23%)		35 (27%)		43 (32%)
Loss of employment		11 (11%)		8 (6%)		11 (8%)
Psychologically abused		8 (7%)		4 (3%)		15 (11%)
Physically abused		0 (0%)		1 (0.8%)		1 (0.7%)
Sexually abused		0 (0%)		0 (0%)		1 (0.8%)
Maintenance of daily routines	3.73 (1.11)		3.46 (1.23)		3.39 (1.21)	
Decreased physical activity	3.13 (1.25)		3.43 (1.27)		3.57 (1.25)	
Decreased social support	1.78 (1.56)		1.95 (1.55)		2.03 (1.56)	
Age	52.9 (10.7)		52.8 (8.5)		51.4 (8.7)	
Number of traumas	—		1.3 (0.5)		1.8 (0.4)	

NT, Non-traumatized group; NIT, Non-interpersonal trauma group; IT, interpersonal trauma group.

No significant differences were found between groups in age, marital status, educational level, pain diagnosis, or pain duration. A significant difference was found between the NIT and IT groups in the number of traumatic events experienced in the past (*t* (263) = 10.90, *p*< 0.001): specifically, more traumatic situations had been experienced by women in the IT group (*M* = 1.76, *SD* = 0.43) than by women in the NIT group (*M* = 1.29, *SD* = 0.46).

In the NIT group, the majority of women (82%) had experienced a life-threatening transportation accident, 77% had experienced a life-threatening illness, and 39% had experienced an unexpected death and/or sudden violent death of a close person. In the IT group, 76% women had experienced a physical assault and 71% had experienced a sexual assault. In addition, 69% women in this group had experienced a life-threatening illness, 66% had experienced a life-threatening transportation accident, and 51% had experienced an unexpected death and/or sudden violent death of a close person.

### Differences Between Groups in Variables Related to COVID-19

The results were as follows: 1) a close relative had been lost to coronavirus by significantly fewer women in the NT group (8%) than by women in the NIT group (χ^2^ (21) = 9.32, *p*< 0.01; Cramer’s V = 0.20) and women in the IT group (19%) (χ^2^ (21) = 5.27, *p*< 0.05; Cramer’s V = 0.15); 2) psychological abuse during lockdown had been experienced by more women in the IT group (11%) than by women in the NIT group (3%) (χ^2^ (1) = 6.42, *p*< 0.05; Cramer’s V = 0.16); and 3) coronavirus had been contracted by more women in the IT group (8%) than by women in the NIT group (2%) (χ2 (1) = 4.52, *p* < 0.05; Cramer’s V = 0.13). No other significant differences were found between the groups. Being physically abused and being sexually abused during lockdown were not included in subsequent analyses due to the low number of cases in the 3 groups of women.

Significant differences with medium effects were found between groups in decreased physical activity (*F*(2,362) = 3.59, *p* = 0.02, *d* = 0.66). The decrease in the level of physical activity during lockdown was less in women in the NT group (post-hoc comparison: *M* = 3.13, *SD* = 1.25) than in the IT group (*M* = 3.57, *SD* = 1.25). No significant differences were found between the NT group and the NIT group or between the NIT group and the IT group. No significant differences between groups were found in maintaining daily routines (*F*(2,362) = 2.56, *p* = 0.08, *d* = 0.51) or decreased social support (*F*(2,362) = 0.74, *p* = 0.47, *d* = 0.18).

### Differences Between Groups in the Variables Pain Interference, Resilience, and Perceived Well-Being

Significant differences between groups were found in pain interference (*F*(2,362) = 5.59, *p*< 0.01, *d* = 0.86). The NT group had lower scores than the IT and NIT groups. No significant differences between groups were found in perceived well-being (*F*(2,362) = 2.65, *p* = 0.07, *d* = 0.53), resilience (*F*(2,362) = 1.07, *p* = 0.34, *d* = 0.24), and pain intensity (*F*(2) = 1.59, *p* = 0.21, *d* = 0.33) (see Table 2).

**TABLE 2:** Mean differences in pain intensity, pain interference, resilience, and perceived well-being in each group (N = 365) (Spain, 2021).

Variables	Group	Mean (SD)	Sum of squares	*F*	*p*	Cohen’s *d*	Post-hoc comparisons
Pain intensity	NT	6.03 (1.73)	942.009	1.59	0.21	0.34	NT-NIT^a^ ^,^ ^b^
	NIT	6.38 (1.49)					NT-IT^b^
	IT	6.11 (1.62)					NIT-IT^b^
Pain interference	NT	40.53 (16.52)	94,531.551	5.59	<0.01	0.86	NT-NIT*
	NIT	46.70 (15.77)					NT-IT**
	IT	46.84 (15.60)					NIT-IT^b^
Perceived well-being	NT	9.36 (4.65)	7,454,575	1.07	0.34	0.24	NT-NIT^b^
	NIT	8.69 (4.36)					NT-IT^b^
	IT	8.52 (4.58)					NIT-IT^b^
Resilience	NT	10.42 (4.60)	7,781.737	2.65	0.07	0.53	NT-NIT^b^
	NIT	9.45 (4.58)					NT-IT^b^
	IT	9.04 (4.62)					NIT-IT^b^

a Bonferroni correction.

b Not significant.

*p < 0.05, **p < 0.01.

NT, Non-traumatized group; NIT, Non-interpersonal trauma group; IT, interpersonal trauma group.

### Univariate General Linear Models for Predicting Pain Interference by Group

For each group, pain interference was considered as the outcome variable, and the following variables were considered as predictors in each model: having contracted COVID-19, having lived with someone with COVID-19, death of a close person due to COVID-19, loss of employment due to COVID-19, access to medical treatment, being psychologically abused, maintenance of daily routines, decreased physical activity, decreased social support, pain intensity, and resilience. We also analysed the pairwise interaction effect of the variables that were found to be significant as well as the total interaction effect when appropriate.

In the NT group, the model proposed for scores on pain-related impairment explained 65% of the variance. The following variables included in the model (i.e., variables that were significant predictors of pain-related impairment) had significant predictive power: pain intensity (ŋ^2^_p_ = 0.57), decreased social support during lockdown (ŋ^2^_p_ = 0.06), and resilience (ŋ^2^_p_ = 0.04). Thus, higher scores on pain related-interference were independently predicted by higher scores on pain intensity and higher levels of loss of social support, whereas lower scores on pain interference predicted higher levels of resilience. The interaction effects were large for pain intensity and decreased social support (ŋ^2^_p_ = 0.18) and for resilience and decreased social support (ŋ^2^_p_ = 0.14), but were medium for pain intensity and resilience (ŋ^2^_p_ = 0.08). A nonsignificant interaction effect was found between pain interference, resilience, and decreased social support. Table 3 shows the model parameters that were statistically significant.

**TABLE 3:** Statistically significant parameters in the GLM for predicting pain interference in the group of women who had never experienced a trauma (*n* = 100), the group of women who had experienced at least one non-interpersonal trauma (*n* = 130), and the group of women who had experienced at least one interpersonal trauma (*n* = 135) (Spain, 2021).

Parameter	Coefficient	SE	*t*	*p*	95% confidence interval		ŋ ^2^_p_
					Lower limit	Upper limit	
NT group							
Decreased social support	1.64	0.65	2.51	0.01	0.342	2.94	0.07
Pain intensity	6.88	0.62	11.19	<0.001	5.66	8.10	0.58
Resilience	−0.47	0.22	−2.18	0.03	−0.901	−0.04	0.05
NIT group							
Pain intensity	4.32	0.78	5.54	<0.001	2.77	5.86	0.20
Resilience	−0.97	0.26	−3.67	<0.001	−1.49	−0.45	0.10
IT group							
Decreased social support	1.82	0.74	2.46	0.02	0.36	3.28	0.05
Pain intensity	3.83	0.70	5.47	<0.001	2.45	5.22	0.19
Resilience	−1.02	0.23	−4.40	<0.001	−1.48	−0.56	0.13

NT, Non-traumatized group; NIT, Non-interpersonal trauma group; IT, Interpersonal trauma group; SE, standard error.

In the NIT group, the model proposed for scores on pain-related impairment explained 34% of the variance. The variables included in this model were pain intensity (ŋ^2^_p_ = 0.21) and resilience (ŋ^2^_p_ = 0.10). Higher levels of pain interference were independently predicted by higher levels of pain intensity. Higher levels of resilience predicted lower scores on pain interference. A nonsignificant interaction effect was found between pain interference and resilience (see Table 3).

In the IT group, the model proposed for scores on pain-related impairment explained 42% of the variance. The variables included in this model were pain intensity (ŋ^2^_p_ = 0.19), resilience (ŋ^2^_p_ = 0.13), and decrease social support (ŋ^2^_p_ = 0.05). Higher scores on pain interference independently predicted higher levels of pain intensity and loss of social support, whereas higher scores on resilience predicted lower scores on pain interference. A significant and medium interaction effect was found for pain intensity and decreased social support (ŋ^2^_p_ = 0.10) (see Table 3).

### Univariate General Linear Models for Predicting Perceived Well-Being by Group

For each group, well-being was considered as the outcome variable. Pain interference and the same variables included in the previous analysis were considered as predictors in each model.

In the NT group, the model proposed for scores on perceived well-being explained 39% of the variance. The following variables included in the model (i.e. variables that were significant predictors of perceived-wellbeing) had significant predictive power: resilience (ŋ^2^_p_ = 0.09), pain interference (ŋ^2^_p_ = 0.07), and maintaining daily routines (ŋ^2^_p_ = 0.06). Higher scores on pain interference independently predicted lower scores on perceived well-being, whereas higher scores in perceived well-being were predicted by higher scores on resilience and on maintaining daily routines. Statistically significant interaction effects were found for pain interference and resilience (ŋ^2^_p_ = 0.10), pain related interference and maintaining daily routines (ŋ^2^_p_ = 0.08), and resilience and maintaining daily routines (ŋ^2^_p_ = 0.08). In all cases, the effects were medium. A nonsignificant interaction effect was found between pain interference, resilience, and maintaining daily routines was not significant. Table 4 shows the model parameters that were statistically significant.

**TABLE 4:** Statistically significant parameters in the GLM for predicting perceived well-being in the in the group of women who had never experienced a trauma (*n* = 100), the group of women who had experienced at least one non-interpersonal trauma (*n* = 130), and the group of women who had experienced at least one interpersonal trauma (*n* = 135) (Spain, 2021).

Parameter	Coefficient	SE	*t*	*p*	95% confidence interval		${ŋ}^{2}$ _p_
					Lower limit	Upper limit	
NT group							
Maintenance of daily routines	0.79	0.34	2.30	0.02	0.11	1.46	0.06
Pain interference	−0.10	0.04	−2.52	0.01	−0.1778	−0.02	0.07
Resilience	0.25	0.09	2.90	0.01	0.78	0.42	0.09
NIT group							
Having contracted COVID-19	−5.75	2.85	−2.02	0.04	−11.39	−0.11	0.03
Maintenance of daily routines	0.71	0.30	2.57	0.015	0.178	1.38	0.05
Decreased social support	−0.52	0.24	−2.19	0.03	−0.98	−0.05	0.04
Pain interference	−0.06	0.03	−2.04	0.04	−0.11	−0.02	0.03
Resilience	0.27	0.08	3.15	0.01	0.10	0.43	0.08
IT group							
Maintenance of daily routines	0.74	0.29	2.50	0.01	0.15	1.32	0.05
Pain interference	−0.07	0.03	−2.60	0.01	−0.13	−0.02	0.05
Resilience	0.28	0.08	3.64	<0.001	0.13	0.44	0.10

NT, Non-traumatized group; NIT, Non-interpersonal trauma group; IT, interpersonal trauma group; SE, standard error.

In the NIT group, the model proposed for scores on perceived well-being explained 31% of the variance. The variables included in this model were resilience (ŋ^2^_p_ = 0.08), maintaining daily routines (ŋ^2^_p_ = 0.05), decreased social support (ŋ^2^_p_ = 0.04), pain interference (ŋ^2^_p_ = 0.03), and having contracted COVID-19 (ŋ^2^_p_ = 0.03). Higher scores on perceived well-being were independently predicted by higher levels of resilience and higher levels of maintaining daily routines, whereas lower scores on perceived well-being were independently predicted by having contracted coronavirus, higher levels of pain interference, and greater loss of social support. The interaction effects were statistically significant and large for resilience and maintenance of daily routines (ŋ^2^_p_ = 0.19), and statistically significant but small for resilience and pain interference (ŋ^2^_p_ = 0.02) (see Table 4).

In the IT group, the model proposed for scores on perceived well-being explained 36% of the variance. The variables included in this model were resilience (ŋ^2^_p_ = 0.10), pain interference (ŋ^2^_p_ = 0.06), and maintaining daily routines (ŋ^2^_p_ = 0.05). Higher levels of pain interference independently predicted lower scores of perceived well-being, whereas higher scores on perceived well-being independently predicted higher scores on resilience and higher levels of maintaining daily routines. A significant and medium interaction effect was found for these 3 variables (ŋ^2^_p_ = 0.05) (see Table 4).

## Discussion

The aim of this study was to investigate the consequences of the COVID-19 lockdown in women with non-malignant CP who had or had not experienced trauma before the outbreak. We also studied differences between the groups in pain interference, resilience, and perceived well-being, and which variables predicted scores on pain interference and perceived well-being in each group. Overall, our results indicate that the worst consequences of the pandemic were experienced by women who had experienced interpersonal traumatic events prior to the COVID-19 outbreak. To our knowledge, this is the first study to have addressed these issues.

The loss of a loved one due to coronavirus or decreased physical activity levels due lockdown are not variables that can be related to previous trauma. Nevertheless, it is not unexpected that more women in the IT group experienced psychological abuse during lockdown, particularly if it is considered that the COVID-19 lockdown has exacerbated relational stressors [24], such as overcrowding, forced cohabitation, and the lack of external social support. Likewise, reports of domestic violence often increase substantially after a catastrophic event [25] and an association has been found between the quarantine mandate and increased intimate partner domestic violence [26]. However, it has also been found that 20% of victims in domestic violence are not the intimate partner but rather a family member or friend [27].

Social support has been hypothesized to have an interaction with both protective and vulnerability factors leading to CP-associated dysfunction [28] and it has been suggested that loneliness and social isolation are the factors that have made this crisis different from other crises [29]. The findings of the present study showed that decreased social support during lockdown was a significant predictor of pain interference in the IT and NT groups, whereas no association was found between these variables in the NIT group. However, it was found that decreased social support was a significant predictor of lower perceived well-being only in the NIT group. This finding may be because more people in this group experienced the death of a close person with COVID-19. Unfortunately, during lockdown, people infected with coronavirus have died alone because the measures to deal with the pandemic have restricted visits from relatives, which has undoubtedly made the situation very difficult for the bereaved. For this reason, it may be the case that the bereaved received more social support (e.g. more expressions of affection from the people with whom they lived), whereas women in the other two groups had less social support because of the restrictions imposed by lockdown.

In contrast to what was predicted, significant differences between groups were only found in the variable pain interference, but not in resilience and perceived well-being. Thus, women who had experienced traumatic events prior to the COVID-19 outbreak showed higher pain interference than women who had never experienced a traumatic event. Traumatic stress is often tied to mental health comorbidities and negative health outcomes such as pain [30]. Thus, when individuals face a mass trauma event, the health consequences can be much more severe, particularly if they have been exposed to past traumatic events. The findings of this study suggest that this is the case. On the other hand, pain intensity and resilience significantly predicted pain interference in the three groups of women. Nevertheless, the percentage of pain interference explained by pain intensity in the NT group was higher than that in the NIT and IT groups, whereas the percentage of pain interference explained by resilience was higher in the NIT and IT groups than in the NT group. This result is in line with previous findings, which have suggested that resilience not only plays a relevant role in CP adjustment [11, 12] but is also of particular value to people who have experienced a traumatic situation before the onset of pain [12]. Resilience is characteristic of people who are able to mobilize psychological resources that they already had before the traumatic event took place. In addition, some studies have found no differences in resilience levels between CP patients who experienced a traumatic event without developing posttraumatic stress symptoms and CP patients who had been never exposed to a traumatic situation [13]. Therefore, it could be speculated that women in these two groups did not have posttraumatic symptomatology. However, these types of symptoms were not addressed in the present study and thus further research is needed on this issue.

Some studies have found that people with CP who report lifetime traumatic events also report greater psychological distress, have more severe physical symptoms, and utilize more health care services [31]. Therefore, we predicted that the lowest statistically significant scores on perceived well-being would be found in women in the IT group. However, this prediction was not fulfilled and so other mediator variables may underlie this finding. For example, depressive and PTSD symptoms have been proposed as relevant pathways by which trauma leads to negative health outcomes [7], while higher levels of anxiety and catastrophizing have been reported in CP patients with a history of lifetime abuse [31]. Unfortunately, none of these variables were addressed in the current study. Nevertheless, the results showed that, in all groups, psychological well-being was predicted by lower levels of pain interference, higher levels of resilience, and maintaining daily routines during lockdown.

The present study has some limitations that limit the generalizability of the results. Firstly, the interpretation of our results could have been substantially affected by possible confounders that were not addressed. Secondly, as this is a cross-sectional study, it was not possible to establish causal effects between the study variables. Thirdly, data collection was exclusively conducted using self-report questionnaires. Fourthly, most of the study participants reported fibromyalgia as their primary pain. Finally, only data obtained from women were analysed.

Disruption of health care during lockdown has only made the situation worse for CP patients and thus, if left untreated, can cause emotional disorders. Therefore, during a crisis of the magnitude of COVID-19, mental health care cannot be separated from other forms of care in these individuals and psychological variables should be taken into account, particularly in traumatized women with CP.

## References

[1] Yu L Kioskli K McCracken LM. The Psychological Functioning in the COVID-19 Pandemic and its Association with Psychological Flexibility and Broader Functioning in People with Chronic Pain. J Pain (2021) 22(8):926–39. 10.1016/j.jpain.2021.02.011 33677112 PMC7930808

[2] Fallon N Brown C Twiddy H Brian E Frank B Nurmikko T Adverse Effects of COVID-19-Related Lockdown on Pain, Physical Activity and Psychological Well-Being in People with Chronic Pain. Br J Pain (2021) 15(3):357–68. 10.1177/2049463720973703 34377461 PMC8339954

[3] Lacasse A Pagé MG Dassieu L Sourial N Janelle-Montcalm A Dorais M Impact of the COVID-19 Pandemic on the Pharmacological, Physical, and Psychological Treatments of Pain: Findings from the Chronic Pain & COVID-19 Pan-Canadian Study. Pain Rep (2021) 6(1):e891. 10.1097/PR9.0000000000000891 33598594 PMC7880148

[4] Serrano-Ibáñez ER Esteve R Ramírez-Maestre C Ruiz-Párraga GT López-Martínez AE. Chronic Pain in the Time of COVID-19: Stress Aftermath and central Sensitization. Br J Health Psychol (2021) 26(2):544–52. 10.1111/bjhp.12483 33099793

[5] Iob E Steptoe A Fancourt D. Abuse, Self-Harm and Suicidal Ideation in the UK during the COVID-19 Pandemic. Br J Psychiatry (2020) 217(4):543–6. 10.1192/bjp.2020.130 32654678 PMC7360935

[6] Shi W Hall BJ. What Can We Do for People Exposed to Multiple Traumatic Events during the Coronavirus Pandemic? Asian J Psychiatr (2020) 51:102065. 10.1016/j.ajp.2020.102065 32298970 PMC7139255

[7] López-Martínez AE Serrano-Ibáñez ER Ruiz-Párraga GT Gómez-Pérez L Ramírez-Maestre C Esteve R Physical Health Consequences of Interpersonal Trauma: A Systematic Review of the Role of Psychological Variables. Trauma Violence Abuse (2018) 19(3):305–22. 10.1177/1524838016659488 27456113

[8] McCall-Hosenfeld JS Winter M Heere T Liebschutz JM. The Association of Interpersonal Trauma with Somatic Symptom Severity in a Primary Care Population with Chronic Pain: Exploring the Role of Gender and the Mental Health Sequelae of Trauma. J Psychosom Res (2014) 77(3):196–204. 10.1016/j.jpsychores.2014.07.011 25149029 PMC4143800

[9] Fishbain D Pulikal A Lewis J Gao J. Chronic Pain Types Differ in Their Reported Prevalence of Post-Traumatic Stress Disorder (PTSD) and there is Consistent Evidence that Chronic Pain Is Associated with PTSD: An Evidence-Based Structured Systematic Review. Pain Med (2017) 18(4):711–35. 10.1093/pm/pnw065 27188666

[10] Bonanno GA Brewin CR Kaniasty K Greca AM. Weighing the Costs of Disaster: Consequences, Risks, and Resilience in Individuals, Families, and Communities. Psychol Sci Public Interest (2010) 11(1):1–49. 10.1177/1529100610387086 26168411

[11] Ramírez-Maestre C Esteve R López-Martínez AE. The Path to Capacity: Resilience and Spinal Chronic Pain. Spine (2012) 37(4):E251–8. 10.1097/BRS.0b013e31822e93ab37 21857397

[12] Ruiz-Párraga GT López-Martínez AE Esteve R Ramírez-Maestre C Wagnild G. A Confirmatory Factor Analysis of the Resilience Scale Adapted to Chronic Pain (RS-18): New Empirical Evidence of the Protective Role of Resilience on Pain Adjustment. Qual Life Res (2015) 24(5):1245–53. 10.1007/s11136-014-0852-z 25377350

[13] Ruiz-Párraga GT López-Martínez AE. The Contribution of Posttraumatic Stress Symptoms to Chronic Pain Adjustment. Health Psychol (2014) 33(9):958–67. 10.1037/hea0000040 24364372

[14] Hamam A Milo S Mor I Shaked E Eliav A Lahav Y Peritraumatic Reactions during the COVID-19 Pandemic-The Contribution of Posttraumatic Growth Attributed to Prior Trauma. J Psychiatr Res (2021) 132:23–31. 10.1016/j.jpsychires.2020.09.029 33038562 PMC7525333

[15] Lahav Y. Psychological Distress Related to COVID-19-The Contribution of Continuous Traumatic Stress. J Affect Disord (2020) 277:129–37. 10.1016/j.jad.2020.07.141 32818776 PMC7416772

[16] Weathers FW Blake DD Schnurr PP Kaloupek DG Marx BP Keane TM. The Life Events Checklist for DSM-5 (LEC-5) (2013). Available from: https://www.ptsd.va.gov/professional/assessment/te-measures/life_events_checklist.asp (Accessed July 8, 2021).

[17] Benjet C Bromet E Karam EG Kessler RC McLaughlin KA Ruscio AM The Epidemiology of Traumatic Event Exposure Worldwide: Results from the World Mental Health Survey Consortium. Psychol Med (2016) 46(2):327–43. 10.1017/S0033291715001981 26511595 PMC4869975

[18] Behling O Law K. Translating Questionnaires and Other Research Instruments: Problems and Solutions. London: SAGE Publications (2000).

[19] Jensen M Karoly P. Self-Report Scales and Procedures for Assessing Pain in Adults. In: Handbook of Pain Assessment. New York: Guilford Press (2001). p. 15–34.

[20] De Andrés-Ares J Cruces L Canos M Penide L Del Valle M Herdman M Validation of the Short Form of the Brief Pain Inventory (BPI-SF) in Spanish Patients with Non-cancer-related Pain. Pain Pract (2015) 15(7):643–53. 10.1111/papr.12219 24766769

[21] Rodríguez-Rey R Alonso-Tapia J Garrido-Hernansaiz H. Reliability and Validity of the Brief Resilience Scale (BRS) Spanish Version. Psychol Assess (2016) 28(5):e101–10. 10.1037/pas0000191 26502199

[22] Lucas-Carrasco R. Reliability and Validity of the Spanish Version of the World Health Organization-Five Well-Being Index in Elderly. Psychiatry Clin Neurosci (2012) 66(6):508–13. 10.1111/j.1440-1819.2012.02387.x 23066768

[23] Cohen J. Statistical Power Analysis for the Behavioral Sciences. 2nd ed. New York: Routlege (1988).

[24] Buttell F Ferreira RJ. The Hidden Disaster of COVID-19: Intimate Partner Violence. Psychol Trauma (2020) 12(S1):S197–S198. 10.1037/tra0000646 32567875

[25] Campbell AM. An Increasing Risk of Family Violence during the Covid-19 Pandemic: Strengthening Community Collaborations to Save Lives. Forensic Sci Int Rep (2020) 2:100089. 10.1016/j.fsir.2020.100089 PMC7152912 38620174

[26] World Health Organization. Coronavirus Disease (COVID-19) Violence against Women (2020). Available from: https://www.who.int/news-room/q-a-detail/coronavirus-disease-covid-19-violence-against-women (Accessed May 12, 2021).

[27] Smith S Fowler K Niolon P. Intimate Partner Homicide and Corollary Victims in 16 States: National Violent Death Reporting System, 2003–2009. Am J Public Health (2014) 104(3):461–6. 10.2105/AJPH.2013.301582 24432943 PMC3953789

[28] Karayannis N Baumann I Sturgeon J Melloh M Mackey S. The Impact of Social Isolation on Pain Interference: A Longitudinal Study. Ann Behav Med (2019) 53(1):65–74. 10.1093/abm/kay017 29668841 PMC6301311

[29] Vinkers CH van Amelsvoort T Bisson JI Branchi I Cryan JF Domschke K Stress Resilience during the Coronavirus Pandemic. Eur Neuropsychopharmacol (2020) 35:12–6. 10.1016/j.euroneuro.2020.05.003 32446705 PMC7211573

[30] Garfin DR Thompson RR Holman EA. Acute Stress and Subsequent Health Outcomes: A Systematic Review. J Psychosom Res (2018) 112:107–13. 10.1016/j.jpsychores.2018.05.017 30097129

[31] Nicol AL Sieberg CB Clauw DJ Hassett AL Moser SE Brummett CM The Association between a History of Lifetime Traumatic Events and Pain Severity, Physical Function, and Affective Distress in Patients with Chronic Pain. J Pain (2016) 17(12):1334–48. 10.1016/j.jpain.2016.09.003 27641311

